# Wastewater monitoring allows the detection of uncommon and highly pathogenic enterovirus types

**DOI:** 10.1128/aem.00534-25

**Published:** 2025-06-23

**Authors:** Lucia Mangeri, Annalisa Scarazzato, Francesco Righi, Elisa Galuppini, Giulia Magagna, Michela Tilola, Virginia Filipello, Enrico Pavoni, Vito Tranquillo, Maria Antonia De Francesco, Marina Nadia Losio, Barbara Bertasi

**Affiliations:** 1 Istituto Zooprofilattico Sperimentale della Lombardia e dell’Emilia-Romagna (IZSLER) 18207 , Brescia, Italy; 2 National Reference Centre for Emerging Risks in Food Safety (CRESA), Istituto Zooprofilattico Sperimentale della Lombardia e dell’Emilia-Romagna (IZSLER) 18207 , Milan, Italy; 3 Institute of Microbiology, Department of Medical, Surgical and Health Sciences, University of Trieste 60267, Trieste, Italy; 4 Institute of Microbiology, Department of Molecular and Translational Medicine, University of Brescia-ASST Spedali Civili 165495, Brescia, Italy; Centers for Disease Control and Prevention, Atlanta, Georgia, USA

**Keywords:** enterovirus, environment, monitoring, wastewater, metabarcoding

## Abstract

The genus *Enterovirus* (EV) includes small ubiquitous viruses with a single-stranded positive-RNA genome. Most EV infections are asymptomatic, but pathogenic EVs are responsible for a wide range of diseases and occasionally epidemics. The aim of the work was to monitor the distribution of EVs present in wastewater collected during the 2 year study period (2022–2023). Three hundred ninety-nine samples were received from three wastewater treatment plants of the Brescia, Cremona, and Bergamo provinces, Lombardy, Italy. One-step real-time reverse transcription-PCR screening showed positivity in 107 samples. Of these, 67 were successfully genotyped: 8 by Sanger sequencing only, 7 by next-generation sequencing metabarcoding only, and 52 by both methods. The most detected species was *Enterovirus B*, followed by *Enterovirus A*, *Enterovirus C,* and *Enterovirus D*. As many as 26 different types of non-polio EVs were identified, of which the most frequently detected were two *Enterovirus B*: echovirus 11 (E11) and coxsackievirus B5 (CVB5). Among *Enterovirus A*, the predominant types were CVA16 and CVA6. As for *Enterovirus C*, the types with the highest frequency were CVA13 and EV-C99; in addition, CVA24, which has been associated with outbreaks of acute hemorrhagic conjunctivitis, was found in two samples from Verziano (Brescia) and one from Bergamo. The *Enterovirus D* species was represented only by type EV-D68, which has recently been related to outbreaks in Europe and the United States. This study reveals a wide EV presence in urban wastewater in the considered area.

**IMPORTANCE:**

Wastewater-based epidemiology uses urban wastewater as a source of dynamic observation for monitoring the circulation of pathogens. A major strength of this surveillance approach is its ability to detect both symptomatic and asymptomatic infections. Through molecular characterization, it was possible to identify uncommon EVs that may lead to serious complications. Furthermore, next-generation sequencing metabarcoding allowed the identification of multiple EV types within a single sample. Wastewater monitoring could therefore be further leveraged as a complementary tool to support the monitoring of severe non-polio EV-related diseases.

## INTRODUCTION

Enteroviruses (EVs) are spread worldwide, causing a broad spectrum of diseases in children and adults, although most EV infections are asymptomatic (1–3). Some types of EV (e.g., EV-A71 and EV-D68) have occasionally been associated with outbreaks that have caused significant morbidity and mortality, as demonstrated in Europe and the United States (4–9). However, EV infections are often underestimated and underdiagnosed (7).

The genus *Enterovirus* (EV) belongs to the family of *Picornaviridae*, which includes viruses with icosahedral symmetry and a 25–30 nm diameter. EVs lack an envelope and have positive single-stranded RNA, 7–8 kb long (10, 11). The genome encodes a large polyprotein that is proteolytically cleaved into four structural proteins (VP1–VP4) forming the viral capsid and seven non-structural proteins (2A–2C, 3A–3D) involved in replication and assembly. The VP1 protein, located on the surface of the capsid, plays a crucial role in receptor binding and immune recognition and exhibits the highest sequence variability among EV types. Molecular typing of EVs relies primarily on sequencing the complete or partial VP1 region. An EV type is defined by sharing less than 75% nucleotide identity and less than 85% amino acid identity with the VP1 sequence of other types (12, 13).

Human EVs are classified into seven species: *Enterovirus A*, *B*, *C*, and *D* (Table 1) and *Rhinovirus A*, *B,* and *C* (3, 14), with more than 270 types of human enteroviruses, divided into polioviruses (PV), coxsackievirus A (CVA), coxsackievirus B (CVB), echovirus (E), enteroviruses (EV), and rhinovirus (RV) (1, 7, 15–17). Only *Enterovirus* A–D species were considered in this study.

**TABLE 1:** Classification of human *Enterovirus A*–*D* species^*a*^

Species	Types
*Enterovirus A* (*n* = 20)	CVA2 → CVA8, CVA10, CVA12, CVA14, CVA16 EV-A71, EV-A76, EV-A89 → EV-A91, EV-A114, EV-A119 → EV-A121
*Enterovirus B* (*n* = 59)	CVA9 CVB1 → CVB6 E1 → E7, E9, E11 → E21, E24 → E27, E29 → E33 EV-B69, EV-B73 → EV-B75, EV-B77 → EV-B88, EV-B93, EV-B97, EV-B98, EV-B100, EV-B101, EV-B106, EV-B107, EV-B111
*Enterovirus C* (*n* = 23)	CVA1, CVA11, CVA13, CVA17, CVA19 → CVA22, CVA24 PV1 → PV3 EV-C95, EV-C96, EV-C99, EV-C102, EV-C104, EV-C105, EV-C109, EV-C113, EV-C116 → EV-C118
*Enterovirus D* (*n* = 4)	EV-D68, EV-D70, EV-D94, EV-D111

^*a*^ Abbreviations: CVA, coxsackievirus A; CVB, coxsackievirus B; EV, enterovirus; E, echovirus; PV, poliovirus.

EVs are ubiquitous, primarily transmitted via the fecal-oral route, and it is facilitated by poor sanitation, overcrowding, ingestion of contaminated food or water, contact with infected fomites (18). After the initial replication in the human intestinal tract, coinciding with the onset of symptoms, EV shedding in feces and the oropharynx can persist for 3–6 weeks in immunocompetent individuals and up to years in immunocompromised people, making environmental shedding from asymptomatic individuals one of the most common ways of virus dissemination (16). The epidemic episodes are mostly observed in semi-closed environments such as schools and kindergartens and predominantly during mild seasons (7). Symptomatic EV infections mainly cause severe disease in children younger than 5 years, premature infants, and immunocompromised people, with clinical manifestations ranging from mild gastrointestinal and respiratory symptoms to more severe conditions like conjunctivitis, hand-foot-mouth disease, meningitis, encephalitis, myocarditis, pericarditis, acute flaccid paralysis (AFP), and inflammatory muscle disease (2). Good hygienic practices, water purification, adequate systems of wastewater disposal, and the control of food at risk of contamination may limit EVs spread and transmission (19, 20).

Wastewater-based epidemiology (WBE) is an approach that exploits urban wastewater as a source of dynamic observation of pathogens circulation. Monitoring of the presence of human enteric viruses in environmental waters began in the 1940s, and it has recently been used to study the circulation of SARS-CoV-2 in the population (18, 21). WBE is a tool for early warning in case of outbreaks, and it is a valuable source of epidemiological data. The intrinsic advantages include the ability to provide objective data, enabling the detection of strains infecting both symptomatic and asymptomatic cases, as well as variations in the spread of pathogens over time and space (7). However, since infected individuals shed the EV until 4 weeks after the peak of infection (up to 10^11^ virus particles/g) and EVs can survive up to 16 weeks in the sewer system, the interpretation of diagnostic results of WBE, particularly when attempting to establish a causal link between viral detection and active disease, can be complicated (2, 7, 16, 18).

Previous environmental surveillance studies of EVs detected both recombinant and vaccine-derived PV (VDPV) strains, but never paralytic or virulent strains (22–24). Poliovirus is becoming a rare cause of AFP worldwide, but non-polio EVs (NPEVs) are on the rise (25). For instance, the types EV-A71 and EV-D68 have emerged as a cause of increasing attention in the pediatric population (8, 16, 26). In Italy, environmental surveillance detected the presence of NPEV and VDPV and their co-circulation and recombination, especially for *Enterovirus C* species. These could give rise to new strains with high virulence, which could represent a potential problem for Public Health (27, 28).

For the routine diagnosis of EV, reverse transcription-PCR (RT-PCR) assays are performed, due to their sensitivity, specificity, and rapidity (15). As for species identification, the sequencing of a region of the capsid protein VP1 gene is considered the gold standard method according to the World Health Organization (1). Should the sequencing of the VP1 region be ineffective, it is recommended to sequence the genes for capsid proteins VP2 and VP4, or the sequencing of the complete genome, if a new recombinant EV is suspected (9). Next-generation sequencing (NGS) allows the simultaneous identification of different microorganisms, thus representing an excellent tool to identify EVs (7). Specifically, in this study, an innovative NGS metabarcoding approach was used to detect different types of EVs in wastewater.

The aim of this study was to monitor the distribution of EVs in wastewater in the Brescia, Cremona, and Bergamo provinces (Lombardy, Italy) and to determine their seasonal dynamics during 2 years of survey. The specific objectives of this study are (i) monitoring the types of EVs known to be associated with severe disease, such as EV-A71 and EV-D68; (ii) confirming the absence of PV; and (iii) monitoring the appearance of new EV types.

## RESULTS

### One-step real-time RT-PCR reactions

EVs were detected in a total of 107 out of 399 samples tested (26.8%). The detailed results of one-step real-time RT-PCR are shown in Table S1a through c in the supplemental material.

**Fig 1:**
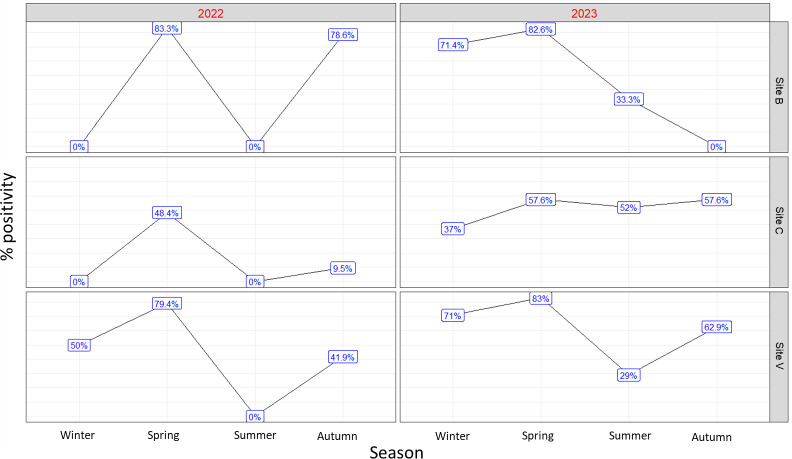
Positivity percentages of enteroviruses detected in wastewater samples from the three sewage treatment plants, during 2022 and 2023. V, Verziano; C, Cremona; and B, Bergamo.

Figure 1 shows EV detection rates in the three wastewater treatment plants (WWTPs), in the four seasons, during 2022 and 2023.

### Sequencing

The semi-nested PCR allowed the amplification of 74 out of the 107 one-step real-time RT-PCR positive samples, which were then sequenced. Of these, 67 were successfully typed: 8 by the Sanger method only, 7 by NGS metabarcoding only, and 52 by both methods. Some samples were sequenced by only one method, likely due to the low concentration of the target in the samples. Detailed results are presented in Table S1a through c in the supplemental material.

With Sanger sequencing, the 60 analyzed samples (GenBank accession numbers of sequences PQ851755 to PQ851814, Table S1a in the supplemental material) were assigned to 16 different EV types: eight (*n* = 45 samples) belonging to *Enterovirus B*, five (*n* = 12 samples) to *Enterovirus A,* and three (*n* = 3 samples) to *Enterovirus C* (5%). The most frequently detected *Enterovirus B* types were echovirus 11 (E11; *n* = 23 samples) and coxsackievirus B5 (CVB5; *n* = 10 samples) (Table 2).

**TABLE 2:** Summary of enterovirus types and number of samples detected by the Sanger method

*Enterovirus A*		*Enterovirus B*		*Enterovirus C*	
Type	Samples (*n*)	Type	Samples (*n*)	Type	Samples (*n*)
CVA4	6	CVB1	1	CVA13	1
CVA5	2	CVB2	5	CVA20	1
CVA6	2	CVB4	1	EV-C99	1
CVA16	1	CVB5	10		
EV-A119	1	E11	23		
		E13	1		
		E18	2		
		E30	2		

Through metabarcoding it was possible to identify 25 EV types on 59 positive samples (GenBank accession numbers available in Table S2b in the supplemental material): *n* = 10 belonging to *Enterovirus B*, *n* = 8 to *Enterovirus A*, *n* = 6 to *Enterovirus C*, and *n* = 1 to *Enterovirus D* (Table 3). Of the 25 types: *n* = 15 were coxsackievirus, *n* = 6 were echovirus, *n* = 4 were enterovirus (EV-A119, EV-A76, EV-C99, EV-D68) and none poliovirus. As shown in Table 3, of the 15 coxsackievirus types, *n* = 10 belonged to type A (CVA) and *n* = 4 belonged to type B (CVB). Finally, five different types were identified among the enteroviruses: *n* = 3 belonging to *Enterovirus A* (EV-A119, EV-A90, and EV-A76), *n* = 1 belonging to *Enterovirus C* (EV-C99), and *n* = 1 belonging to *Enterovirus D* (EV-D68).

**TABLE 3:** Qualitative and quantitative summary of *Enterovirus* types detected in samples sequenced by the NGS metabarcoding method

*Enterovirus A*		*Enterovirus B*		*Enterovirus C*		*Enterovirus D*	
Type	No. reads	Type	No. reads	Type	No. reads	Type	No. reads
CVA4	40,766	CVB1	24,103	CVA1	2,144	EV-D68	18
CVA5	131,591	CVB2	234,052	CVA13	71,910		
CVA6	53,700	CVB4	5,334	CVA19	13		
CVA10	1,583	CVB5	333,331	CVA22	1,051		
CVA16	73,987	E3	149	CVA24	2,647		
EV-A119	258	E11	1,357,106	EV-C99	29,441		
EV-A76	43,206	E13	15,919	PV 1-3	0		
EV-A90	4,922	E18	46,214	VDPV	0		
		E21	81,679				
		E30	96,661				

At the quantitative level, based on the total number of reads obtained by metabarcoding, excluding results with fewer than 10 reads and contig size below 275 bp (*n* = 2,651,785), the following distributions were observed: 83% *Enterovirus B* (*n* = 2,194,562 reads), 13% *Enterovirus A* (*n* = 349,999 reads), 4% *Enterovirus C* (*n* = 107,206 reads), and 0.001% *Enterovirus D* (*n* = 18 reads).

**Fig 2:**
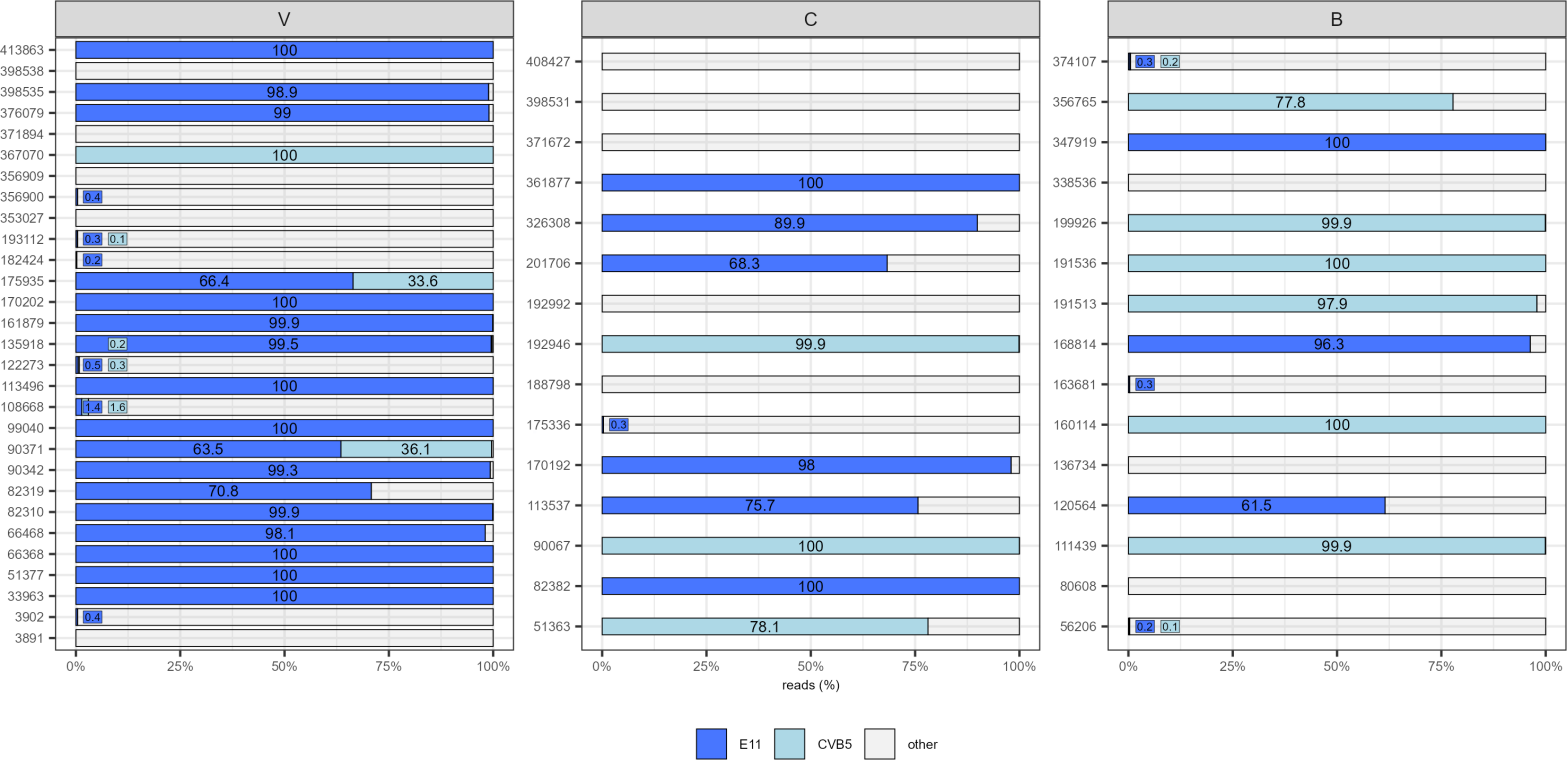
Distributions of the two most frequently detected enterovirus types in 59 samples sequenced by NGS metabarcoding. E, echovirus; CVB, coxsackievirus B; V, Verziano (Brescia); C, Cremona; and B, Bergamo.

The 2,651,785 reads resulted in type identification: echoviruses accounted for 37% (*n* = 976,212), coxsackieviruses for 60% (*n* = 1,597,728), and enteroviruses for 3% (*n* = 77,845). Among coxsackieviruses, 61% belonged to type B (*n* = 596,820) and 39% to type A (*n* = 379,392). Within CVBs, the most frequently encountered types were CVB5 with 56% (*n* = 333,331) and CVB2 with 39% (*n* = 234,052). Of the CVAs, the major representatives were 35% CVA5 (*n* = 131,591), 20% CVA16 (*n* = 73,987), 14% CVA6 (*n* = 53,700), and 14% CVA4 (*n* = 53,700), all belonging to the *Enterovirus A* species. Among echoviruses, E11 was the most prevalent with 85% (*n* = 1,357,106). Fig. 2 shows the distributions of the two most frequently detected EV types (E11 and CVB5); the representation is based on read counts, expressed as percentages. Among the five different enterovirus types, the most frequently encountered were EV-A76, accounting for 56% (*n* = 43,206), and EV-C99, accounting for 38% (*n* = 29,441) (Table 3).

In conclusion, considering both Sanger and NGS sequencing methods, as many as 26 different types of NPEVs were detected in this work.

**Fig 3:**
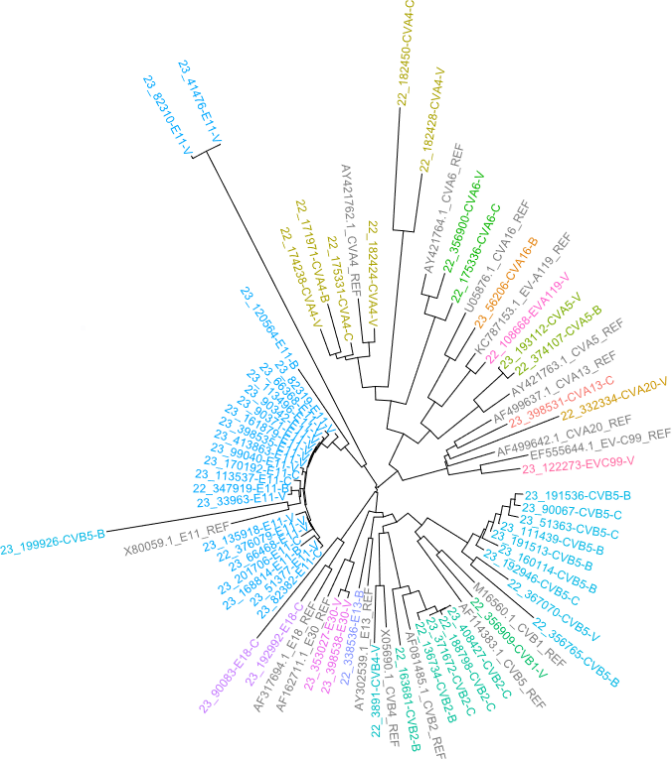
Phylogenetic tree of sequences obtained through the Sanger method from 60 samples. CVA, coxsackievirus A; CVB, coxsackievirus B; EV, enterovirus; E, echovirus; REF, reference sequence. Sample origins: Verziano (Brescia); C, Cremona; and B, Bergamo. Different colors indicate different enterovirus types.

**Fig 4:**
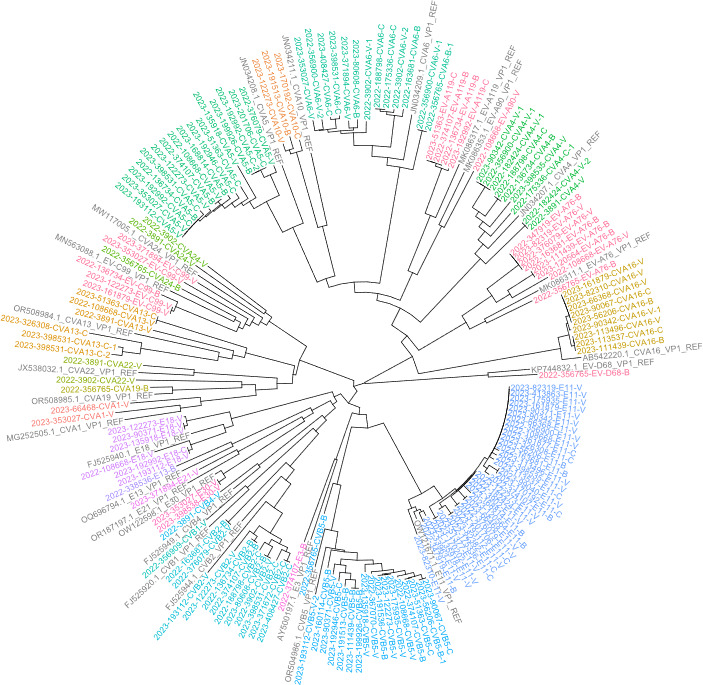
Phylogenetic tree of sequences obtained through NGS metabarcoding from 59 samples. CVA, coxsackievirus A; CVB, coxsackievirus B; EV, enterovirus; E, echovirus; REF, reference sequence. Sample origins: V, Verziano (Brescia); C, Cremona; B, Bergamo. Different colors indicate different enterovirus types.

The phylogenetic tree in Fig. 3 shows the sequences obtained by the Sanger method (*n* = 60 sample sequences and *n* = 16 reference sequences), while Fig. 4 shows the sequences obtained by NGS metabarcoding of 59 samples (*n* = 162 sample sequences and *n* = 25 reference sequences).

**Fig 5:**
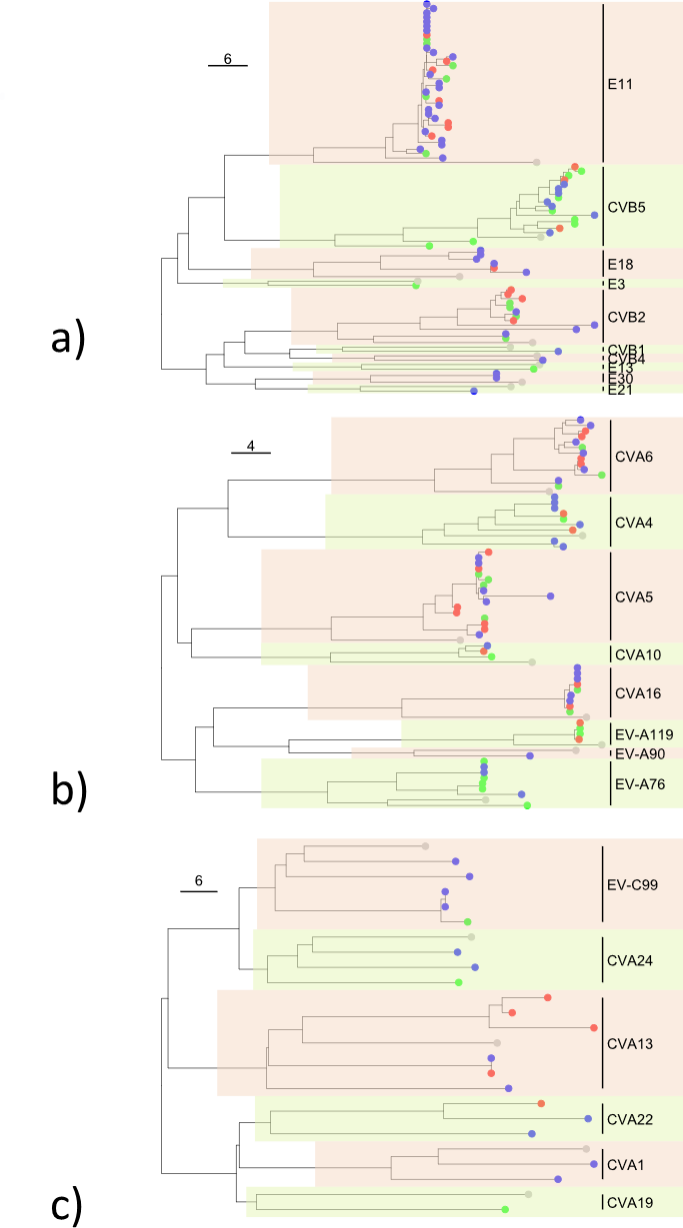
Phylogenetic trees of the 59 samples sequenced by NGS metabarcoding method, grouped by species as follows. (**a**) *Enterovirus A*; (**b**) *Enterovirus B*; and (**c**) *Enterovirus C*. CVA, coxsackievirus A; CVB, coxsackievirus B; EV, enterovirus; and E, echovirus. Different colors represent sample origins: blue for Verziano (Brescia), red for Cremona, green for Bergamo, and grey for reference sequences.

Figure 5 shows the phylogenetic trees grouped by species, based on the same sequences obtained by NGS metabarcoding: *Enterovirus A* (*n* = 63 sample sequences and *n* = 8 reference sequences), *Enterovirus B* (*n* = 79 sample sequences and *n* = 10 reference sequences), and *Enterovirus C* (*n* = 19 sample sequences and *n* = 6 reference sequences). *Enterovirus D* species, being represented by only one type, was not included.

**Fig 6:**
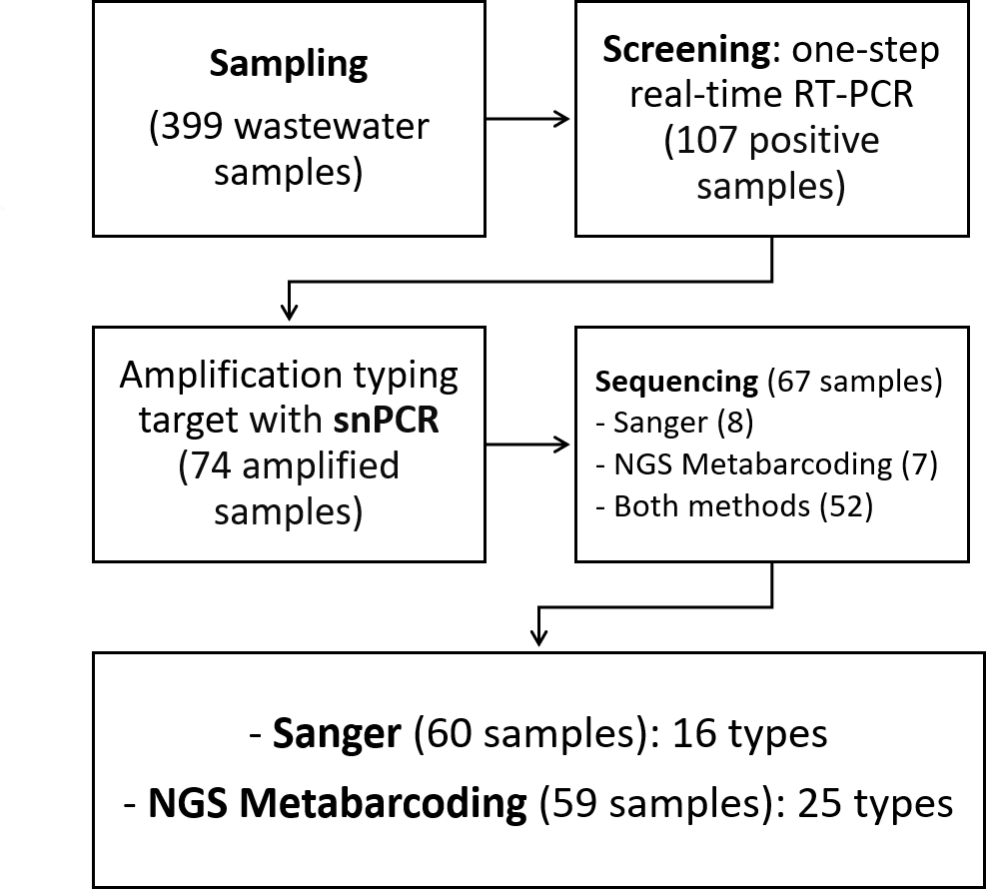
Flowchart showing the number of wastewater samples processed in this study, from initial collection to the final number of successfully sequenced samples.

Finally, Fig. 6 illustrates the flowchart of sample results.

## DISCUSSION

EVs are spread worldwide. *Enterovirus B* is the most detected species, while the other species show continent-specific differences, with *Enterovirus A* being the most detected in Asia and *Enterovirus C* the most detected in Africa (3). In this work, the most detected species was *Enterovirus* B, followed by *Enterovirus* A, C, and D. This trend was observed by both Sanger (*Enterovirus B*, 75%; *Enterovirus A*, 20%; *Enterovirus C*, 5%) and metabarcoding methods, considering the reads counts (*Enterovirus B*, 83%; *Enterovirus A*, 13%; *Enterovirus C*, 4%; and *Enterovirus D*, 0.001%). These data agree with what was recently reported by the National Enterovirus Surveillance System (NESS, United States), which also defined *Enterovirus B* as the most frequent species, followed by *Enterovirus A*, *Enterovirus C,* and *Enterovirus D* (7).

In this study, a total of 26 different NPEV types were identified. As already reported in other studies, in Italy, there is a high rate of NPEV detection in wastewater samples collected before the purification process, demonstrating the widespread presence of these viruses in the population (2, 28–33). In addition, it has been reported that the efficiency of purification systems in abating viral load, particularly EVs, is not complete, underscoring the need to introduce virological parameters, alongside bacteriological ones, to verify the safety of effluent leaving from the purification systems (31).

In this work, EVs were detected in 26.8% of wastewater samples. This percentage is lower than other Italian studies, reporting different sample numbers and positivity rates (*n* = 188, 79%; *n* = 321, 67%; *n* = 21, 62% and *n* = 2,880, 40–56%; *n* = 33, 67%) (30–33). Nevertheless, the use of process control, which allowed for validating adequate recovery efficiency, increased the robustness of this study.

The most frequently detected EV types were two members of the *Enterovirus B* species: E11 and CVB5, being the most represented *Enterovirus B* types, accounting for 62 and 15% of the total *Enterovirus B* reads, respectively (Fig. 2; Table 3). These results agree with the distribution of EVs detected in wastewater samples in northern Italy from 2009 to 2015 and with two recent Chinese studies (31, 34, 35). The higher detection of CVB could be due to its endemic pattern causing a constant presence in wastewater. Regarding *Enterovirus A* species, the most spread types in the world are: CVA6, CVA16, and EV-A71 (3). In this study, CVA16 and CVA6 are well represented, accounting for 20 and 14% of the total *Enterovirus A* reads. Moreover, within *Enterovirus A,* a prominent type was CVA5, accounting for 35% of the total *Enterovirus A* reads. Regarding the *Enterovirus C* species, EV-C99 and CVA13 were those with the highest frequency, even though in literature they are mainly reported in Africa (3). Indeed, EV-C99 accounted for 23% of the total *Enterovirus C* reads, CVA13 (11%) and CVA24 (1%). It is relevant to mention that CVA24 has been associated with outbreaks of hemorrhagic conjunctivitis in different parts of the world and only occasionally observed in environmental surveillance (36). Therefore, the identification of types belonging to *Enterovirus C* species is particularly important due to their ability to recombine with PVs, potentially leading to the emergence of highly virulent novel strains (27, 28).

The *Enterovirus D* species is represented only by type EV-D68, which is generally detected in respiratory samples but was previously detected in municipal wastewater, although in very low concentration and sporadically (3, 7). EV-D68 shows a distinctive biennial circulation pattern, with peaks typically observed every 2 years. This periodicity is likely driven by fluctuating population immunity and viral evolution (37).

Finally, it emerged from this work that PV is not circulating in the Lombardy region. Through the National Health Institute surveillance of AFP and environmental circulation of PV and other EVs in Italy, only one Sabin-like PV was detected and isolated before, which was likely imported from children vaccinated abroad with Sabin vaccine or their adult contacts (24).

The results obtained from the present monitoring are consistent with those reported in the 2013 ISTISAN Reports 13/44 (ISS). In Italy, in 2011, E6 (28%) was the most frequently identified echovirus, followed by E7 (26%), E11 (23%), and E3 (13%), and, among the coxsackieviruses, CVB5 (54%) followed by CVB2 (22%) (24).

It should be noted that the samples from the three different WWTPs were highly heterogeneous; on average, 18 different types of EVs were identified per WWTP (*n* = 21 Verziano—Brescia; *n* = 11 Cremona; *n* = 17 Bergamo), out of a total of 26 types found. The distribution of EVs, at species level (A–C), is comparable between the different WWTPs, as shown in Fig. 5. Furthermore, the phylogenetic trees generated with all sequences clustered the samples consistently to reference sequences and by type (Fig. 3 and 4). The only exceptions were sample 2023/199926, identified in the GenBank database as CVB5 following Sanger sequencing, which clustered with the reference sequence of E11 (Fig. 3); and the samples 2022/356765 and 2022/3902 sequencing, identified in the GenBank database as CVA24 and CVA22 following NGS metabarcoding, which clustered with the reference sequence of EV-C99 and CVA19, respectively (Fig. 4).

Several studies showed that *Enterovirus A* and *Enterovirus B* exhibit seasonality, with *Enterovirus B* implicated in more than 80% of cases during the summer and fall months and *Enterovirus A* accounting for 45% of cases in spring (38). Conversely, *Enterovirus C* is observed throughout the year, with a peak most likely in the winter period, while *Enterovirus D* shows variable and sporadic trends (3, 39, 40). However, epidemic episodes due to EV infections are predominantly observed during mild seasons (7, 19, 20). In fact, data provided by the NESS (United States), collected from 1970 to 2013, revealed a consistent spike in EV infections from June to October (7). This seasonal pattern was also found in this study: the percentage of EV-positive samples increased in the spring and in the autumn in both 2022 and 2023 (Fig. 1). Unfortunately, due to the limited number of positive samples, it was not possible to perform a statistically robust quantitative comparison.

For monitoring EV circulation in wastewater, the viral isolation approach using cell culture is widely used, followed by RT-semi-nested PCR (RT-snPCR) amplification and Sanger sequencing of the VP1 gene. However, the cell culture method may introduce bias and selective imbalance for some EV strains, recovered from the environment (35). This issue is relevant especially in complex sample types, such as municipal sewage, where multiple types of EVs are frequently co-circulating. In addition, some EV types, such as CVA19, require inoculation in mice to be propagated and do not result in any cytopathic effect on the cell culture. Consequently, several types of EVs (generally *Enterovirus B*) might have been detected more frequently, while others more rarely (34, 35). Confirming this, CVA19 was found in this work, albeit in a single sample and at a low concentration (*n* = 13 reads; 0.03% of total *Enterovirus C*), showing the usefulness of NGS approaches to improve our knowledge on the circulation of less common types.

Recent studies have shown that NGS is an excellent tool for monitoring the diversity of enteric viruses, such as EVs, adenoviruses, noroviruses, and astroviruses in wastewater, offering greater sensitivity than Sanger sequencing and enabling the detection of multiple strains and potentially virulent recombinants (7, 34, 35, 41–44).

In this study, metabarcoding enabled the identification of an average of three different EV types per sample, with up to seven types in two samples from the Verziano (Brescia) WWTP. Notably, the sequence variant with the most reads detected by NGS matched the Sanger results in 100% of cases at the species level and 99.9% at the type level, with the only exception being sample 2022/108668. This innovative NGS metabarcoding approach proved to be an effective and efficient molecular tool for detecting multiple EV types in wastewater. Despite these advantages, molecular testing presents some limitations, including the inability to assess viral viability, potential PCR inhibition from organic matter or metals in environmental samples, and reduced sensitivity to low viral loads, particularly during high flow rates at WWTPs, for instance during heavy rainfalls (28, 29). In addition, sequencing outcomes are influenced by factors such as viral recovery efficiency, viral concentration methods, and primer performance (34).

At the technical level, nucleotide identity percentages, based on identification through the GenBank database, for sequences obtained using the Sanger method ranged from 72.7 to 98.9%, with an average of 91.7%. For those detected by NGS metabarcoding, identity ranged from 82.8 to 100.0%, with an average of 96.1%. Only one sequence (2022-3891-CVA13-V) was analyzed using the Enterovirus Genotyping Tool due to a low match in the GenBank database.

In Europe, the European Non-Polio Enterovirus network plays a key role in improving the diagnosis of EV infections, collecting epidemiological data, and monitoring the circulation of EV types. This platform facilitates timely diagnosis and promotes the sharing of information across the community (9). Given the significant gap between the estimated numbers of asymptomatic infections and reported clinical cases, it is essential to integrate clinical trends with environmental monitoring (43). Investigating EVs in wastewater in relation to clinical cases or outbreaks offers a valuable model for understanding the epidemiology of intestinal viral pathogens at the population level (29). Several studies have emphasized the importance of enhanced surveillance, rapid outbreak detection, and the implementation of preventive measures, due to the potential severity of EV-related complications (45, 46).

Unfortunately, the absence of clinical data on EV infections and hospitalizations in the area covered by this study prevented the assessment of any correlation between EV types detected in wastewater and those causing clinical illness. To address this, future efforts should focus on establishing a parallel surveillance system targeting patients with symptoms suggestive of EV infection.

### Conclusions

This study revealed that EVs are widely present in urban wastewater in the analyzed area, that they are heterogeneous (encompassing all four A–D species), and that they show apparent seasonal peaks. The combination of consistent temporal sampling of wastewater, efficient virus concentration procedures, and NGS analysis enabled the detection of a broad spectrum of EV types. Moreover, molecular characterization confirmed the circulation of less commonly detected EVs, including some typical respiratory strains, which could lead to severe complications, such as CVA24 and EV-D68. NGS sequencing proved to be a valuable tool, with potential applications for monitoring other viral pathogens in wastewater, such as norovirus, hepatitis E virus, hepatitis A virus, influenza, and adenovirus.

The application of NGS technology in WBE can significantly enhance our knowledge on EV circulation patterns in the population. It may serve as a complementary procedure to clinical surveillance, which currently operates on an individual basis. WBE, by contrast, enables the monitoring of the entire population, offering insights into the spread and persistence of specific viral strains and supporting the development of predictive epidemiological models.

## MATERIALS AND METHODS

### Sampling

The samples were collected through the official SARS-CoV-2 surveillance coordinated by the National Health Institute (ISS).

Water sampling was performed at the inlet of the WWTPs of Verziano (Brescia), Cremona, and Bergamo, prior to depuration treatments. These plants treat up to 290,000, 180,000, and 200,000 inhabitant equivalents (IE) respectively, where IE is defined as “the biodegradable organic load having a 5-day biochemical oxygen demand (BOD5) of 60 grams of oxygen per day*”* (47). Sampling was performed between January 2022 and December 2023 and was carried out by the operators of the sewage treatment plants, when possible, twice a week for the Verziano and Cremona plants and once a week in Bergamo. Automatic samplers were used to obtain the 24 hour composite average in a volume of 1 L. Composite average sampling consists of the collection of multiple samplings at defined intervals of time throughout 24 hours. All samplings are collected and homogenized in a tank from which the water bottles conferred to the laboratory are filled. After that, the wastewater containers were put at refrigerated temperature for up to 48 hours before analysis, or at −20°C for longer storage periods.

In total, the laboratory received 399 samples: 177 from Verziano (Brescia), 160 from Cremona, and 62 from Bergamo.

### Sample preparation and nucleic acid extraction

The first steps of the analysis, including viral inactivation, concentration, and transfer to the extraction lysis buffer, were performed under a class two cabinet, in a biocontainment laboratory.

After a thermal pre-treatment at 56°C for 30 minutes, in a water bath to inactivate any infectious viral particles, 50 mL of sample was transferred to a sterile tube, and 100 µL of recombinant Mengovirus (10^2^ TCID_50_/mL, viral process control provided by Superior Institute of Health, Rome, Italy) was added as process control. The samples were spun at 4,500 × *g* for 30 minutes at 4°C in a centrifuge with anti-aerosol safety closure, without brake (brake 0). Then, 45 mL of supernatant was gently added to 0.9 g NaCl and 4 g polyethylene glycol 8000 (PEG_8000_). The tubes were then mildly agitated for 15 minutes at 4°C to completely dissolve the salts and centrifuged at 12,000 × *g* for 120 minutes at 4°C. The supernatant was discarded, and the pellet was centrifuged again at 12,000 × *g* for 5 minutes at 4°C. Finally, the remaining supernatant was completely discarded, and each pellet was resuspended in 500 µL PBS.

The viral RNA was extracted using the eGENE-UP Lysis and RNA/DNA Purification kit (bioMérieux, Marcy-l'Etoile—France), following the manufacturer’s directions, and stored at −80°C until use.

### One-step real-time RT-PCR reactions

EV detection was carried out by one-step real-time RT-PCR with primers targeting the noncoding region at the 5′ (48) (Table 4). For process control, a Mengovirus-specific one-step real-time RT-PCR was set up (21).

**TABLE 4:** Primer and probe sets used for the Enterovirus screening and Mengovirus one-step real-time PCR

		Sequence (5′–3′)
Enterovirus		
Primer_rhient-For EV	Forward	CCTCCGGCCCCTGA
Primer_P1.4taq-Rev EV	Reverse	GATTGTCACCATAAGCAGCC
Probe_Entpr1	Probe	FAM-CGGAACCGACTACTTTGGGT-TAMRA
Mengovirus		
Primer_Mengo 110 For	Forward	GCGGGTCCTGCCGAAAGT
Primer_Mengo 209 Rev	Reverse	GAAGTAACATATAGACAGACGCACAC
Probe_Mengo 147	Probe	FAM-ATCACATTACTGGCCGAAGC-MGB

EV one-step real-time RT-PCR was performed with Invitrogen RNA UltraSense One-Step Quantitative RT-PCR System kit (Thermo Fisher Scientific, Waltham, MA, USA) in a total volume of 25  µL, containing 11.25 µL of DNAsi/RNAsi free water, 5 µL of 5 × Ultrasense Reaction Mix, 1  µL of each primer (10 µM; rhient-For EV and P1.4taq-Rev EV), 0.5  µL of Entpr1 probe (10 µM), 1.25  µL of RNA Ultrsense Enzyme Mix, and 5  µL of extracted viral RNA.

The thermal profile of the reaction was 50°C for 20 minutes (reverse transcription; RT), followed by an initial denaturation step at 95°C for 5 minutes and 45 cycles of 15 seconds at 95°C and 1 minute at 60°C.

Mengovirus one-step real-time RT-PCR was performed with the same kit in a total volume of 25 µL, containing 9.62 µL of DNAsi/RNAsi free water, 5 µL of 5× Ultrasense Reaction Mix, 1.25 µL of Mengo 110 For primer (10 µM), 2.25 µL of Mengo 209 Rev primer (10 µM), 0.63  µL of Mengo 147 probe (10 µM), 1.25  µL of RNA Ultrsense Enzyme Mix, and 5 µL of extracted viral RNA.

The thermal profile of the reaction was: 55°C for 60 minutes (RT), followed by an initial denaturation step at 95°C for 5 minutes and 45 cycles of 15 seconds at 95°C, 1 minute at 60°C, and 1 minute at 65°C (21). All reactions were performed on a CFX96 Touch Real-Time PCR Detection System (Bio-Rad, Hercules, CA, USA).

The analysis of results was done by evaluating the performance of the samples Ct, in relation to the standard curve of the Mengovirus. For process verification, the analysis was considered valid according to La Rosa et al. (21).

### Sequencing

For EV typing, a region of the VP1 gene was sequenced.

#### RT-snPCR for VP1 amplification

The RNA RT was performed with FIREScript KIT (Solis BioDyne, Tartu, Estonia), in a total volume of 20  µL, containing 5.5 µL of DNAsi/RNAsi free water, 2  µL of 10×  Transcriptase Buffer, 1  µL dNTPs pool (10  mM), 5  µL of cocktail primers (40  µM, Table 5), 0.5  µL of RNAse Inhibitor (40  U/µL; Promega, Madison, WI, USA), 1  µL of MMLV Reverse Transcriptase (200  U/µL), and 5  µL of extracted viral RNA. The cocktail primers consisted of a pool of AN32, AN33, AN34, and AN35 primers (1). RT was carried out at 22°C for 10 minutes, followed by 42°C for 30 minutes, and a step at 94°C for 5 minutes.

**TABLE 5:** Primer sets used for RT-snPCR for VP1 amplification, Sanger, and NGS metabarcoding genotyping

	Primer direction	Sequence (5′–3′)
Retrotranscription		
AN32	Reverse	GTYTGCCA
AN33	Reverse	GAYTGCCA
AN34F	Reverse	CCRTCRTA
AN35	Reverse	RCTYTGCCA
First PCR		
SO224 F	Forward	GCIATGYTIGGIACICAYRT
SO222 R	Reverse	CICCIGGIGGIAYRWACAT
Semi-nested PCR and Sanger genotyping		
AN89 F	Forward	CCAGCACTGACAGCAGYNGARAYNGG
AN88 R	Reverse	TACTGGACCACCTGGNGGNAYRWACAT
NGS metabarcoding genotyping		
AN232 F metabar	Forward	TCGTCGGCAGCGTCAGATGTGTATAAGAGACAGCCAGC ACTGACAGCA
AN233 R metabar	Reverse	GTCTCGTGGGCTCGGAGATGTGTATAAGAGACAGTACT GGACCACCTGG

The first PCR was performed in a total volume of 25 µL containing 12.5 µL of 2× HotStart ReadyMix (KAPA HiFi; Kapa Biosystems, Wilmington, MA, USA), 2.5 µL of each specific primer (10 µM) (Table 5) (1), 2.5 µL of DNAse-RNAse-free water and 5 µL of cDNA. The PCR reaction was performed with the following thermal profile: 15 minutes at 95°C, followed by 35 cycles of 30 seconds at 94°C, 30 seconds at 42°C, 0.4°C/s until 60°C, 45 seconds at 60°C, and a final extension at 72°C for 10 minutes.

Lastly, a semi-nested PCR was set up in a total volume of 25 µL containing 12.5 µL of 2× HotStart ReadyMix (KAPA HiFi), 1.25 µL of each primer (10 µM) (Table 5) (1), 5 µL of DNAse-RNAse-free water, and 5 µL of the first PCR template. The amplification conditions were 15 minutes at 95°C, followed by 35 cycles of 30 seconds at 94°C, 1 minute at 60°C, 1 minute at 72°C, and a final extension at 72°C for 10 minutes.

All reactions were performed on a ProFlex PCR Thermal Cycler System (Applied Biosystems, Carlsbad, CA, USA) and according to Enterovirus Surveillance Guidelines (1).

The analysis of semi-nested PCR products was performed by capillary electrophoresis (expected target between 348 and 393 bp) on a QIAxcel Advanced System (Qiagen GmbH, Hilden, Germany) using the QIAxcel DNA Screening Cartridge (Qiagen GmbH).

#### Genotyping by Sanger method

The amplicons were purified using ExoSAP-IT Express PCR Product Cleanup Reagent (Thermo Fisher Scientific, Waltham, MA, USA), according to the manufacturer’s instructions.

The forward and reverse sequence reactions were performed separately using the primers listed in Table 5. Each reaction mix was prepared in a total volume of 10 µL containing 2 µL of 2.5 × Big Dye Terminator Reaction Mix (Thermo Fisher Scientific), 1 µL of 5 × Big Dye Terminator Sequencing Buffer, 3 µL of DNAse-RNase-free water, 2 µL of the corresponding primer (1.6 µM), and 2 µL of purified product. Samples were incubated at 96°C for 1 minute and amplified for 25 cycles at 96°C for 10 seconds, 50°C for 5 seconds, and 60°C for 4 minutes, on a ProFlex Thermal Cycler. The reaction products were purified using the BigDye XTerminator Purification Kit (Thermo Fisher) according to the manufacturer’s instructions. Samples were then sequenced on a SeqStudio Genetic Analyzer (Applied Biosystems, Foster City, CA, USA).

The consensus sequences were generated with Molecular Evolutionary Genetics Analysis Version 6.0 (MEGA6) software (49) and used for identifying the species and types of EV present in the sample through the Nucleotide GenBank database using the Basic Local Alignment Search Tool (BLAST) (https://blast.ncbi.nlm.nih.gov/Blast.cgi).

#### Next-Generation Sequencing (NGS) metabarcoding on the Illumina MiSeq platform

The semi-nested PCR products were normalized at a concentration of 5 ng/µL, following quantification using the QuantiFluor ONE dsDNA kit and a Quantifluor fluorometer (Promega, Madison, Wisconsin, USA). Then, an initial PCR with primers modified with overhang adapter sequences was performed. The reaction was prepared in a total volume of 25 µL, containing 12.5 µL of 2 × HotStart ReadyMix (KAPA HiFi), 5 µL of each primer (1 µM) (Table 5), and 2.5 µL of the normalized PCR product under the following conditions: 3 minutes at 95°C, 25 cycles of 30 seconds at 95°C, 30 seconds at 55°C, 30 seconds at 72°C, and a final extension at 72°C for 5 minutes.

PCR products were then purified using the Agencourt AMPure XP kit (Beckman Coulter Genomics, Brea, CA, USA). For the preparation of sequencing libraries, dual indexes (Nextera XT Index Kit, Illumina, San Diego, CA, USA) were ligated, and a second purification of the PCR products was performed according to the Guide “16S Metagenomic Sequencing Library Preparation” (50) provided by Illumina (San Diego, CA, USA).

This was followed by quantification of the sequencing libraries and quality control with the QIAxcel Advanced System equipped with a Qiaxcel DNA High Resolution cartridge. The libraries were considered suitable if they showed a fragment distribution profile between 495 and 540 bp. The normalization and pooling of the libraries were performed following the Guide “16S Metagenomic Sequencing Library Preparation” (50). Sequencing was performed on the MiSeq—Illumina System (–Illumina, San Diego, CA, USA) using a MiSeq Reagent Nano Kit v2 (500 cycles) following the manufacturer’s instructions. The running parameters evaluated were Cluster Density (1,000–1,200 K/mm^2^), Clusters Passing Filter (>75%), percentage of bases with Q-Score > 30, and Error Rate PhiX (<6%). Samples were considered suitable after evaluation of the quality report provided by the FastQC module of the Genome Detective Virus Tool (https://www.genomedetective.com/app/typingtool/virus/) (51) that trims and discards sequences of lower quality using predetermined criteria.

For each sample, fastq files R1 and R2 were submitted to the Genome Detective Platform (https://www.genomedetective.com/db/ui/submit), selecting the protocol “Default Viral Analysis” and the sequencing technology “Illumina PE (Paired-End).” Finally, the platform generated a report of the identified species and types, including the number of reads, nucleotide identity, genome coverage, and correlated sequences. Sequences with more than 10 reads and a length greater than 275 bp were then confirmed for species and type identification using the Nucleotide GenBank database via the BLAST (https://blast.ncbi.nlm.nih.gov/Blast.cgi).

### Creation of phylogenetic trees

Evolutionary analyses and phylogenetic tree construction were performed using MEGA6 software (49), with tree visualization and annotation carried out using the ggtree package (https://bioconductor.org/packages/release/bioc/html/ggtree.html). The evolutionary history was inferred using the Neighbor-Joining method (52).

The reference sequences were retrieved from the International Committee on Taxonomy of Viruses Official Taxonomic Resources (https://ictv.global/report/chapter/picornaviridae/picornaviridae/enterovirus) for Fig. 3 and 5 and from GenBank for Fig. 4.

### Statistical analysis

Given the non-probabilistic nature of the sampling adopted, the results are presented, in tabular and graphical form as frequencies (%), and as far as the NGS metabarcoding method is concerned, reads are considered as counts.

## Data Availability

The nucleotide sequences determined in this study were deposited in the GenBank database with accession numbers available in Table S2a in the supplemental material.

## SUPPLEMENTAL MATERIAL

Tables S1 and S2 aem.00534-25-s0001.docx Results of typing and GenBank accession numbers.

ASM does not own the copyrights to Supplemental Material that may be linked to, or accessed through, an article. The authors have granted ASM a non-exclusive, world-wide license to publish the Supplemental Material files. Please contact the corresponding author directly for reuse.
