# Supplementary material for: Wastewater monitoring allows the detection of uncommon and highly pathogenic enterovirus types
Source: Appl Environ Microbiol. 2025 Jun 23;91(7):e00534-25. doi: 10.1128/aem.00534-25 (PMC12285228; doi:10.1128/aem.00534-25)
Supplement: Tables S1 and S2 — Results of typing and GenBank accession numbers. [file aem.00534-25-s0001.docx]

**TABLE S1a.** Results of typing with Sanger method and metabarcoding (NGS) of the 33 sequenced samples from the Verziano (Brescia) sewage treatment plant*.* CVA, coxsackievirus A; CVB, coxsackievirus B; EV, enterovirus; E, echovirus

| **ID** | **Ct one-step real-time RT-PCR** | **Sanger species** | **Sanger type** | **% identity*** | **NGS species** | **NGS types** | **% identity*** | **no. reads** | **Contig/Contigs size (bp)** |
| --- | --- | --- | --- | --- | --- | --- | --- | --- | --- |
| **2022/3891** | 39*.*61 | *Enterovirus B* | CVB4 | 95*.*4 | *Enterovirus B* | CVB4 | 98.0 | 5334 | 346 |
|  |  |  |  |  | *Enterovirus C* | CVA13 | 81.7** | 1772 | 351 |
|  |  |  |  |  | *Enterovirus A* | CVA4 | 94.5 | 477 | 322 |
|  |  |  |  |  | *Enterovirus C* | CVA22 | 99.4 | 30 | 348 |
|  |  |  |  |  | *Enterovirus C* | CVA24 | 83.4 | 26 | 351 |
| **2022/3902** | 36*.*52 | / | / | / | *Enterovirus A* | CVA6 | 99.1 - 98.7 | 3277 | 321 - 322 |
|  |  |  |  |  | *Enterovirus C* | CVA24 | 83*.*3 | 1160 | 353 |
|  |  |  |  |  | *Enterovirus C* | CVA22 | 93.1 | 1021 | 363 |
|  |  |  |  |  | *Enterovirus B* | E11 | 99.4 | 20 | 345 |
|  |  |  |  |  | *Enterovirus B* | CVB2 | 98.8 | 11 | 348 |
| **2022/108668** | ≥ 40*.*00 | *Enterovirus A* | EV-A119 | 93*.*0 | *Enterovirus A* | EV-A90 | 95.6 | 4922 | 323 |
|  |  |  |  |  | *Enterovirus B* | CVB5 | 97.7 | 82 | 342 |
|  |  |  |  |  | *Enterovirus B* | E11 | 99.7 | 71 | 345 |
|  |  |  |  |  | *Enterovirus A* | CVA5 | 97.1 | 32 | 321 |
|  |  |  |  |  | *Enterovirus B* | E18 | 93.0 | 22 | 348 |
|  |  |  |  |  | *Enterovirus C* | CVA13 | 83.4 | 18 | 354 |
|  |  |  |  |  | *Enterovirus A* | EV-A76 | 93.0 | 14 | 321 |
| **2022/174238** | 31*.*68 | *Enterovirus A* | CVA4 | 92*.*1 | / | / | / | / | / |
| **2022/182424** | 32*.*01 | *Enterovirus A* | CVA4 | 91*.*2 | *Enterovirus A* | CVA4 | 95.5 -95.7 | 32462 | 276 - 276 |
|  |  |  |  |  | *Enterovirus B* | E11 | 99.4 | 49 | 345 |
| **2022/182428** | 32*.*39 | *Enterovirus A* | CVA4 | 96*.*3 | / | / | / | / | / |
| **2022/332334** | 37*.*11 | *Enterovirus C* | CVA2O | 77*.*0 | / | / | / | / | / |
| **2022/356900** | 36*.*73 | *Enterovirus A* | CVA6 | 98*.*9 | *Enterovirus A* | CVA6 | 95.8 - 97*.*0 | 16116 | 323 - 338 |
|  |  |  |  |  | *Enterovirus B* | E11 | 99.7 | 65 | 348 |
|  |  |  |  |  | *Enterovirus A* | CVA4 | 94.6 | 27 | 321 |
| **2022/356909** | 34*.*99 | *Enterovirus B* | CVB1 | 89*.*3 | *Enterovirus B* | CVB1 | 98.4 | 24103 | 366 |
| **2022/367070** | 34*.*68 | *Enterovirus B* | CVB5 | 83*.*7 | *Enterovirus B* | CVB5 | 97.1 | 46721 | 348 |
| **2022/376079** | 33*.*01 | *Enterovirus B* | E11 | 94*.*0 | *Enterovirus B* | E11 | 97.9 | 55073 | 373 |
|  |  |  |  |  | *Enterovirus A* | CVA5 | 99.2 | 507 | 389 |
|  |  |  |  |  | *Enterovirus B* | CVB2 | 95.6 | 17 | 348 |
|  |  |  |  |  | *Enterovirus A* | EV-A76 | 96.9 | 17 | 322 |
| **ID** | **Ct one-step real-time RT-PCR** | **Sanger species** | **Sanger type** | **% identity*** | **NGS species** | **NGS types** | **% identity*** | **no. reads** | **Contig/Contigs size (bp)** |
| **2023/33963** | ≥ 40*.*00 | *Enterovirus B* | E11 | 96*.*7 | *Enterovirus B* | E11 | 97.2 | 81198 | 363 |
| **2023/41476** | 39*.*28 | *Enterovirus B* | E11 | 95*.*2 | / | / | / | / | / |
| **2023/51377** | ≥ 40*.*00 | *Enterovirus B* | E11 | 94*.*7 | *Enterovirus B* | E11 | 97.8 | 49642 | 386 |
| **2023/66368** | ≥ 40*.*00 | *Enterovirus B* | E11 | 97*.*5 | *Enterovirus B* | E11 | 99.2 | 58490 | 361 |
|  |  |  |  |  | *Enterovirus A* | CVA16 | 97.2 | 21 | 324 |
| **2023/66468** | ≥ 40*.*00 | *Enterovirus B* | E11 | 94*.*0 | *Enterovirus B* | E11 | 97.6 | 29684 | 386 |
|  |  |  |  |  | *Enterovirus C* | CVA1 | 95.3 | 563 | 348 |
| **2023/82310** | ≥ 40*.*00 | *Enterovirus B* | E11 | 93*.*1 | *Enterovirus B* | E11 | 97.5 | 36739 | 362 |
|  |  |  |  |  | *Enterovirus A* | CVA16 | 96.9 | 28 | 324 |
| **2023/82319** | ≥ 40*.*00 | *Enterovirus B* | E11 | 93*.*0 | *Enterovirus B* | E11 | 97.9 | 43096 | 376 |
|  |  |  |  |  | *Enterovirus A* | EV-A76 | 96.9 | 17733 | 326 |
| **2023/90342** | ≥ 40*.*00 | *Enterovirus B* | E11 | 95*.*9 | *Enterovirus B* | E11 | 98.9 | 43799 | 387 |
|  |  |  |  |  | *Enterovirus A* | CVA16 | 98.1 | 256 | 325 |
|  |  |  |  |  | *Enterovirus A* | CVA4 | 94.6 | 36 | 321 |
| **2023/90371** | ≥ 40*.*00 | *Enterovirus B* | E11 | 96*.*1 | *Enterovirus B* | E11 | 99.7 | 23118 | 347 |
|  |  |  |  |  | *Enterovirus B* | CVB5 | 98.5 | 13116 | 344 |
|  |  |  |  |  | *Enterovirus B* | E18 | 98.5 | 146 | 349 |
| **2023/99040** | 37*.*08 | *Enterovirus B* | E11 | 96*.*6 | *Enterovirus B* | E11 | 97.7 | 131710 | 361 |
| **2023/113496** | 39*.*63 | *Enterovirus B* | E11 | 98*.*0 | *Enterovirus B* | E11 | 98.1 | 73537 | 366 |
|  |  |  |  |  | *Enterovirus A* | CVA16 | 97.5 | 26 | 324 |
| **2023/122273** | 38*.*64 | *Enterovirus C* | EV-C99 | 82*.*8 | *Enterovirus C* | EV-C99 | 84.7 | 24280 | 367 |
|  |  |  |  |  | *Enterovirus B* | E11 | 99.1 | 127 | 345 |
|  |  |  |  |  | *Enterovirus B* | CVB5 | 97.6 | 69 | 348 |
|  |  |  |  |  | *Enterovirus B* | E18 | 97.4 | 63 | 348 |
|  |  |  |  |  | *Enterovirus A* | CVA10 | 97.8 | 29 | 321 |
|  |  |  |  |  | *Enterovirus A* | CVA5 | 92.7 | 21 | 321 |
|  |  |  |  |  | *Enterovirus B* | CVB2 | 89.8 | 20 | 348 |
| **2023/135918** | 36*.*21 | *Enterovirus B* | E11 | 97*.*3 | *Enterovirus B* | E11 | 97.7 | 42404 | 353 |
|  |  |  |  |  | *Enterovirus B* | E18 | 95.9 | 109 | 349 |
|  |  |  |  |  | *Enterovirus B* | CVB5 | 98.0 | 85 | 342 |
|  |  |  |  |  | *Enterovirus A* | CVA5 | 98.2 | 11 | 457 |
| **2023/161879** | 33*.*42 | *Enterovirus B* | E11 | 98*.*0 | *Enterovirus B* | E11 | 97.8 | 26939 | 361 |
|  |  |  |  |  | *Enterovirus A* | CVA16 | 97.5 | 23 | 324 |
|  |  |  |  |  | *Enterovirus C* | EV-C99 | 84.7 | 12 | 351 |
| **ID** | **Ct one-step real-time RT-PCR** | **Sanger species** | **Sanger type** | **% identity*** | **NGS species** | **NGS types** | **% identity*** | **no. reads** | **Contig/Contigs size (bp)** |
| **2023/170202** | 32*.*49 | / | / | / | *Enterovirus B* | E11 | 96.7 | 28913 | 364 |
| **2023/175935** | 35*.*46 | / | / | / | *Enterovirus B* | E11 | 99.1 | 140 | 345 |
|  |  |  |  |  | *Enterovirus B* | CVB5 | 98.0 | 71 | 342 |
| **2023/193112** | 32*.*24 | *Enterovirus A* | CVA5 | 93*.*5 | *Enterovirus A* | CVA5 | 97.7 | 43583 | 275 |
|  |  |  |  |  | *Enterovirus B* | E11 | 99.4 | 110 | 345 |
|  |  |  |  |  | *Enterovirus B* | CVB5 | 96.1 | 60 | 356 |
|  |  |  |  |  | *Enterovirus B* | E18 | 99.1 | 37 | 348 |
|  |  |  |  |  | *Enterovirus B* | CVB2 | 94.8 | 11 | 348 |
| **2023/353027** | 31*.*61 | *Enterovirus B* | E30 | 94*.*6 | *Enterovirus B* | E30 | 94.8 | 46752 | 381 |
|  |  |  |  |  | *Enterovirus A* | CVA5 | 97.8 | 5419 | 336 |
|  |  |  |  |  | *Enterovirus C* | CVA1 | 98.2 | 1581 | 337 |
|  |  |  |  |  | *Enterovirus C* | EV-C99 | 87.6 | 1243 | 351 |
|  |  |  |  |  | *Enterovirus A* | CVA6 | 98.1 | 135 | 262 |
| **2023/371894** | 30*.*83 | / | / | / | *Enterovirus B* | E21 | 89.1 | 81679 | 350 |
|  |  |  |  |  | *Enterovirus C* | EV-C99 | 87.3 | 3881 | 353 |
|  |  |  |  |  | *Enterovirus A* | CVA6 | 96.6 | 1591 | 323 |
| **2023/398535** | 31*.*96 | *Enterovirus B* | E11 | 97*.*3 | *Enterovirus B* | E11 | 98.9 | 90261 | 352 |
|  |  |  |  |  | *Enterovirus A* | CVA4 | 97.4 | 1003 | 263 |
| **2023/398538** | 35*.*55 | *Enterovirus B* | E30 | 93*.*7 | *Enterovirus B* | E30 | 96.1 | 49909 | 366 |
| **2023/413863** | 35*.*19 | *Enterovirus B* | E11 | 97*.*5 | *Enterovirus B* | E11 | 98.4 | 207380 | 366 |

* percentage of identity detected with BLAST® (GenBank)

** percentage of identity matched with Enterovirus Genotyping Tool

**TABLE S1b.** Results of typing with Sanger method and metabarcoding (NGS) of the 18 sequenced samples from the Cremona sewage treatment plant*.* CVA, coxsackievirus A; CVB, coxsackievirus B; EV, enterovirus; E, echovirus

| **ID** | **Ct one-step real-time RT-PCR** | **Sanger species** | **Sanger type** | **% identity*** | **NGS species** | **NGS types** | **% identity*** | **no. reads** | **Contig/Contigs size (bp)** |
| --- | --- | --- | --- | --- | --- | --- | --- | --- | --- |
| **2022/175331** | 35*.*87 | *Enterovirus A* | CVA4 | 97*.*8 | / | / | / | / | / |
| **2022/175336** | 38*.*39 | *Enterovirus A* | CVA6 | 95*.*0 | *Enterovirus A* | CVA6 | 98.4 | 19866 | 349 |
|  |  |  |  |  | *Enterovirus A* | CVA4 | 98.4 | 6112 | 337 |
|  |  |  |  |  | *Enterovirus B* | E11 | 98.8 | 72 | 345 |
| **2022/182450** | 32*.*88 | *Enterovirus A* | CVA4 | 95*.*5 | / | / | / | / | / |
| **ID** | **Ct one-step real-time RT-PCR** | **Sanger species** | **Sanger type** | **% identity*** | **NGS species** | **NGS types** | **% identity*** | **no. reads** | **Contig/Contigs size (bp)** |
| **2022/188798** | 36*.*07 | *Enterovirus B* | CVB2 | 94*.*2 | *Enterovirus B* | CVB2 | 97.8 | 9069 | 363 |
|  |  |  |  |  | *Enterovirus A* | CVA6 | 98.8 | 2141 | 321 |
|  |  |  |  |  | *Enterovirus A* | CVA4 | 96.3 | 514 | 336 |
| **2023/51363** | ≥ 40*.*00 | *Enterovirus B* | CVB5 | 93*.*7 | *Enterovirus B* | CVB5 | 97.3 | 36334 | 344 |
|  |  |  |  |  | *Enterovirus C* | CVA13 | 83.3 | 10125 | 355 |
|  |  |  |  |  | *Enterovirus A* | EV-A119 | 93.9 | 61 | 321 |
|  |  |  |  |  | *Enterovirus A* | CVA5 | 93.6 | 23 | 321 |
| **2023/82382** | ≥ 40*.*00 | *Enterovirus B* | E11 | 94*.*6 | *Enterovirus B* | E11 | 98.8 | 65562 | 347 |
| **2023/90067** | ≥ 40*.*00 | *Enterovirus B* | CVB5 | 94*.*9 | *Enterovirus B* | CVB5 | 97.0 | 41787 | 344 |
|  |  |  |  |  | *Enterovirus A* | CVA16 | 97.5 | 15 | 324 |
| **2023/90083** | ≥ 40*.*00 | *Enterovirus B* | E18 | 92*.*8 | / | / | / | / | / |
| **2023/113537** | 39*.*34 | *Enterovirus B* | E11 | 96*.*7 | *Enterovirus B* | E11 | 98.6 | 104827 | 361 |
|  |  |  |  |  | *Enterovirus A* | CVA16 | 95.6 | 33559 | 340 |
| **2023/170192** | 32*.*73 | *Enterovirus B* | E11 | 97*.*6 | *Enterovirus B* | E11 | 98.0 | 34884 | 361 |
|  |  |  |  |  | *Enterovirus A* | CVA10 | 97.4 | 695 | 355 |
| **2023/192946** | 34*.*55 | *Enterovirus B* | CVB5 | 95*.*2 | *Enterovirus B* | CVB5 | 94.5 | 37439 | 370 |
|  |  |  |  |  | *Enterovirus A* | CVA5 | 96.2 | 52 | 321 |
| **2023/192992** | 33*.*25 | *Enterovirus B* | E18 | 96*.*0 | *Enterovirus B* | E18 | 94.9 | 45837 | 377 |
|  |  |  |  |  | *Enterovirus A* | EV-A119 | 93.2 | 25 | 321 |
|  |  |  |  |  | *Enterovirus A* | CVA5 | 96.8 - 98.1 | 38 | 321 - 321 |
| **2023/201706** | 32*.*38 | *Enterovirus B* | E11 | 94*.*9 | *Enterovirus B* | E11 | 98.3 | 24515 | 361 |
|  |  |  |  |  | *Enterovirus A* | CVA5 | 99.0 | 11374 | 333 |
| **2023/326308** | ≥ 40*.*00 | / | / | / | *Enterovirus B* | E11 | 98.8 | 116 | 345 |
|  |  |  |  |  | *Enterovirus C* | CVA13 | 85.5 | 13 | 354 |
| **2023/361877** | 35*.*71 | / | / | / | *Enterovirus B* | E11 | 98.3 | 103 | 345 |
| **2023/371672** | ≥ 40*.*00 | *Enterovirus B* | CVB2 | 98*.*0 | *Enterovirus B* | CVB2 | 97.5 | 74586 | 363 |
| **2023/398531** | 30*.*19 | *Enterovirus C* | CVA13 | 89*.*6 | *Enterovirus C* | CVA13 | 91.9 - 89.5 | 59982 | 385 - 354 |
|  |  |  |  |  | *Enterovirus A* | CVA5 | 97.7 | 22301 | 323 |
|  |  |  |  |  | *Enterovirus A* | CVA6 | 98.4 | 3115 | 323 |
|  |  |  |  |  | *Enterovirus B* | CVB2 | 97.9 | 99 | 349 |
| **2023/408427** | ≥ 40*.*00 | *Enterovirus B* | CVB2 | 97*.*9 | *Enterovirus B* | CVB2 | 96.2 | 84616 | 375 |
|  |  |  |  |  | *Enterovirus A* | CVA6 | 98.7 | 5115 | 323 |

* percentage of identity detected with BLAST® (GenBank)

**TABLE S1c.** Results of typing with Sanger method and metabarcoding (NGS) of the 16 sequenced samples from the Bergamo sewage treatment plant*.* CVA, coxsackievirus A; CVB, coxsackievirus B; EV, enterovirus; E, echovirus

| **ID** | **Ct one-step real-time RT-PCR** | **Sanger species** | **Sanger type** | **% identity*** | **NGS species** | **NGS types** | **% identity*** | **no. reads** | **Contig size (bp)** |
| --- | --- | --- | --- | --- | --- | --- | --- | --- | --- |
| **2022/136734** | ≥ 40*.*00 | *Enterovirus B* | CVB2 | 95*.*2 | *Enterovirus B* | CVB2 | 97.5 | 27963 | 372 |
|  |  |  |  |  | *Enterovirus A* | CVA4 | 96.9 | 135 | 321 |
|  |  |  |  |  | *Enterovirus A* | CVA5 | 97.2 | 69 | 321 |
|  |  |  |  |  | *Enterovirus A* | EV-A119 | 93.1 | 42 | 321 |
|  |  |  |  |  | *Enterovirus C* | EV-C99 | 82.8 | 25 | 351 |
| **2022/163681** | 35*.*97 | *Enterovirus B* | CVB2 | 90*.*4 | *Enterovirus B* | CVB2 | 95.3 | 12277 | 350 |
|  |  |  |  |  | *Enterovirus A* | CVA6 | 99.1 | 961 | 323 |
|  |  |  |  |  | *Enterovirus B* | E11 | 100.0 | 39 | 345 |
|  |  |  |  |  | *Enterovirus A* | EV-A76 | 96.8 | 13 | 280 |
| **2022/171971** | 32*.*97 | *Enterovirus A* | CVA4 | 87*.*5 | / | / | / | / | / |
| **2022/338536** | 35*.*12 | *Enterovirus B* | E13 | 88*.*8 | *Enterovirus B* | E13 | 88.9 | 15919 | 347 |
| **2022/347919** | 36*.*15 | *Enterovirus B* | E11 | 98*.*5 | *Enterovirus B* | E11 | 98.3 | 39279 | 361 |
|  |  |  |  |  | *Enterovirus A* | EV-A76 | 97.1 | 11 | 321 |
| **2022/356765** | 36*.*86 | *Enterovirus B* | CVB5 | 72*.*7 | *Enterovirus B* | CVB5 | 87.7 | 5412 | 353 |
|  |  |  |  |  | *Enterovirus C* | CVA24 | 83.4 | 1461 | 305 |
|  |  |  |  |  | *Enterovirus A* | EV-A76 | 93.9 | 40 | 321 |
|  |  |  |  |  | *Enterovirus D* | EV-D68 | 98.7 | 18 | 363 |
|  |  |  |  |  | *Enterovirus A* | CVA6 | 95.6 | 15 | 321 |
|  |  |  |  |  | *Enterovirus C* | CVA19 | 98.5 | 13 | 348 |
| **2022/374107** | 33*.*32 | *Enterovirus A* | CVA5 | 95*.*9 | *Enterovirus A* | CVA5 | 97.2 | 47169 | 323 |
|  |  |  |  |  | *Enterovirus B* | E3 | 98.5 | 149 | 345 |
|  |  |  |  |  | *Enterovirus B* | E11 | 97.7 | 134 | 345 |
|  |  |  |  |  | *Enterovirus A* | EV-A119 | 92.8 | 130 | 321 |
|  |  |  |  |  | *Enterovirus B* | CVB5 | 97.3 | 114 | 330 |
|  |  |  |  |  | *Enterovirus B* | CVB2 | 98.2 | 20 | 348 |
| **2023/56206** | ≥ 40*.*00 | *Enterovirus A* | CVA16 | 96*.*2 | *Enterovirus A* | CVA16 | 96.4 | 40046 | 340 |
|  |  |  |  |  | *Enterovirus B* | E11 | 100.0 | 100 | 345 |
|  |  |  |  |  | *Enterovirus B* | CVB5 | 96.7 | 22 | 334 |
| **2023/80608** | 37*.*61 | / | / | / | *Enterovirus B* | CVB2 | 97.3 | 25363 | 376 |
|  |  |  |  |  | *Enterovirus A* | CVA6 | 99.3 | 1368 | 303 |
| **2023/111439** | 39*.*30 | *Enterovirus B* | CVB5 | 95*.*6 | *Enterovirus B* | CVB5 | 96.2 | 37920 | 338 |
|  |  |  |  |  | *Enterovirus A* | EV-A76 | 94.7 | 14 | 321 |
| **ID** | **Ct one-step real-time RT-PCR** | **Sanger species** | **Sanger type** | **% identity*** | **NGS species** | **NGS types** | **% identity*** | **no. reads** | **Contig size (bp)** |
|  |  |  |  |  | *Enterovirus A* | CVA16 | 97.4 | 13 | 324 |
| **2023/120564** | 35*.*20 | *Enterovirus B* | E11 | 90*.*7 | *Enterovirus B* | E11 | 98.8 | 40449 | 347 |
|  |  |  |  |  | *Enterovirus A* | EV-A76 | 94.5 | 25364 | 337 |
| **2023/160114** | 30*.*27 | *Enterovirus B* | CVB5 | 94*.*3 | *Enterovirus B* | CVB5 | 95.6 | 11153 | 350 |
| **2023/168814** | 32*.*68 | *Enterovirus B* | E11 | 86*.*9 | *Enterovirus B* | E11 | 99.1 | 24461 | 358 |
|  |  |  |  |  | *Enterovirus A* | CVA5 | 98.4 | 943 | 340 |
| **2023/191513** | 32*.*75 | *Enterovirus B* | CVB5 | 96*.*2 | *Enterovirus B* | CVB5 | 96.3 | 39548 | 338 |
|  |  |  |  |  | *Enterovirus A* | CVA10 | 98.4 | 859 | 321 |
| **2023/191536** | 32*.*09 | *Enterovirus B* | CVB5 | 96*.*0 | *Enterovirus B* | CVB5 | 97.1 | 29767 | 349 |
| **2023/199926** | 32*.*12 | *Enterovirus B* | CVB5 | 98*.*7 | *Enterovirus B* | CVB5 | 98.1 | 33631 | 344 |
|  |  |  |  |  | *Enterovirus A* | CVA5 | 98.4 | 49 | 322 |

* percentage of identity detected with BLAST® (GenBank)

**TABLE S2a.** GenBank accession numbers of sequences obtained by the Sanger method*.* CVA, coxsackievirus A; CVB, coxsackievirus B; EV, enterovirus; E, echovirus

| **ID sequence** | **Accession number** | **Species** | **Type** |
| --- | --- | --- | --- |
| 22_3891 | PQ851755 | *Enterovirus B* | CVB4 |
| 22_108668 | PQ851756 | *Enterovirus A* | EV-A119 |
| 22_136734 | PQ851757 | *Enterovirus B* | CVB2 |
| 22_163681 | PQ851758 | *Enterovirus B* | CVB2 |
| 22_171971 | PQ851759 | *Enterovirus A* | CVA4 |
| 22_174238 | PQ851760 | *Enterovirus A* | CVA4 |
| 22_175331 | PQ851761 | *Enterovirus A* | CVA4 |
| 22_175336 | PQ851762 | *Enterovirus A* | CVA6 |
| 22_182424 | PQ851763 | *Enterovirus A* | CVA4 |
| 22_182428 | PQ851764 | *Enterovirus A* | CVA4 |
| 22_182450 | PQ851765 | *Enterovirus A* | CVA4 |
| 22_188798 | PQ851766 | *Enterovirus B* | CVB2 |
| 22_332334 | PQ851767 | *Enterovirus C* | CVA2O |
| 22_338536 | PQ851768 | *Enterovirus B* | E13 |
| 22_347919 | PQ851769 | *Enterovirus B* | E11 |
| 22_356765 | PQ851770 | *Enterovirus B* | CVB5 |
| 22_356900 | PQ851771 | *Enterovirus A* | CVA6 |
| 22_356909 | PQ851772 | *Enterovirus B* | CVB1 |
| 22_367070 | PQ851773 | *Enterovirus B* | CVB5 |
| 22_374107 | PQ851774 | *Enterovirus A* | CVA5 |
| 22_376079 | PQ851775 | *Enterovirus B* | E11 |
| 23_33963 | PQ851776 | *Enterovirus B* | E11 |
| 23_41476 | PQ851777 | *Enterovirus B* | E11 |
| 23_51363 | PQ851778 | *Enterovirus B* | CVB5 |
| 23_51377 | PQ851779 | *Enterovirus B* | E11 |
| 23_56206 | PQ851780 | *Enterovirus A* | CVA16 |
| 23_66368 | PQ851781 | *Enterovirus B* | E11 |
| 23_66468 | PQ851782 | *Enterovirus B* | E11 |
| 23_82310 | PQ851783 | *Enterovirus B* | E11 |
| 23_82319 | PQ851784 | *Enterovirus B* | E11 |
| 23_82382 | PQ851785 | *Enterovirus B* | E11 |
| 23_90067 | PQ851786 | *Enterovirus B* | CVB5 |
| 23_90083 | PQ851787 | *Enterovirus B* | E18 |
| 23_90342 | PQ851788 | *Enterovirus B* | E11 |
| 23_90371 | PQ851789 | *Enterovirus B* | E11 |
| 23_99040 | PQ851790 | *Enterovirus B* | E11 |
| 23_111439 | PQ851791 | *Enterovirus B* | CVB5 |
| 23_113496 | PQ851792 | *Enterovirus B* | E11 |
| 23_113537 | PQ851793 | *Enterovirus B* | E11 |
| 23_120564 | PQ851794 | *Enterovirus B* | E11 |
| 23_122273 | PQ851795 | *Enterovirus C* | EV-C99 |
| 23_135918 | PQ851796 | *Enterovirus B* | E11 |
| 23_160114 | PQ851797 | *Enterovirus B* | CVB5 |
| 23_161879 | PQ851798 | *Enterovirus B* | E11 |
| 23_168814 | PQ851799 | *Enterovirus B* | E11 |
| 23_170192 | PQ851800 | *Enterovirus B* | E11 |
| 23_191513 | PQ851801 | *Enterovirus B* | CVB5 |
| 23_191536 | PQ851802 | *Enterovirus B* | CVB5 |
| 23_192946 | PQ851803 | *Enterovirus B* | CVB5 |
| 23_192992 | PQ851804 | *Enterovirus B* | E18 |
| 23_193112 | PQ851805 | *Enterovirus A* | CVA5 |
| 23_199926 | PQ851806 | *Enterovirus B* | CVB5 |
| 23_201706 | PQ851807 | *Enterovirus B* | E11 |
| 23_353027 | PQ851808 | *Enterovirus B* | E30 |
| 23_371672 | PQ851809 | *Enterovirus B* | CVB2 |
| 23_398531 | PQ851810 | *Enterovirus C* | CVA13 |
| 23_398535 | PQ851811 | *Enterovirus B* | E11 |
| 23_398538 | PQ851812 | *Enterovirus B* | E30 |
| 23_408427 | PQ851813 | *Enterovirus B* | CVB2 |
| 23_413863 | PQ851814 | *Enterovirus B* | E11 |

**TABLE S2b.** GenBank accession numbers of sequences obtained by the NGS metabarcoding*.* CVA, coxsackievirus A; CVB, coxsackievirus B; EV, *enterovirus*; E, echovirus

| **ID sequence** | **Accession number** | **Species** | **Type** |
| --- | --- | --- | --- |
| 2022-3891-CVA13-V | PV006242 | *Enterovirus C* | CVA13 |
| 2022-3891-CVA22-V | PV006243 | *Enterovirus C* | CVA22 |
| 2022-3891-CVA4-V | PV006244 | *Enterovirus A* | CVA4 |
| 2022-3891-CVB4-V | PV006245 | *Enterovirus B* | CVB4 |
| 2022-3902-CVA24-V | PV006246 | *Enterovirus C* | CVA24 |
| 2022-3902-CVA6-V-1 | PV006248 | *Enterovirus A* | CVA6 |
| 2022-3902-CVA6-V-2 | PV006249 | *Enterovirus A* | CVA6 |
| 2022-3902-CVB2-V | PV006251 | *Enterovirus B* | CVB2 |
| 2022-3902-E11-V | PV006254 | *Enterovirus B* | E11 |
| 2022-108668-CVA13-V | PV006257 | *Enterovirus C* | CVA13 |
| 2022-108668-CVB5-V | PV006259 | *Enterovirus B* | CVB5 |
| 2022-108668-E11-V | PV006260 | *Enterovirus B* | E11 |
| 2022-108668-E18-V | PV006261 | *Enterovirus B* | E18 |
| 2022-136734-CVA4-B | PV006263 | *Enterovirus A* | CVA4 |
| 2022-136734-CVA5-B | PV006264 | *Enterovirus A* | CVA5 |
| 2022-136734-CVB2-B | PV006265 | *Enterovirus B* | CVB2 |
| 2022-136734-EVA119-B | PV006266 | *Enterovirus A* | EV-A119 |
| 2022-136734-EVC99-B | PV006267 | *Enterovirus C* | EV-C99 |
| 2022-163681-CVA6-B | PV006269 | *Enterovirus A* | CVA6 |
| 2022-163681-CVB2-B | PV006270 | *Enterovirus B* | CVB2 |
| 2022-163681-E11-B | PV006271 | *Enterovirus B* | E11 |
| 2022-163681-EVA76-B | PV006272 | *Enterovirus A* | EV-A76 |
| 2022-175336-CVA4-C-1 | PV006273 | *Enterovirus A* | CVA4 |
| 2022-175336-CVA6-C | PV006275 | *Enterovirus A* | CVA6 |
| 2022-175336-E11-C | PV006276 | *Enterovirus B* | E11 |
| 2022-182424-CVA4-V-1 | PV006277 | *Enterovirus A* | CVA4 |
| 2022-182424-CVA4-V-2 | PV006278 | *Enterovirus A* | CVA4 |
| 2022-182424-E11-V | PV006280 | *Enterovirus B* | E11 |
| 2022-188798-CVA4-C | PV006281 | *Enterovirus A* | CVA4 |
| 2022-188798-CVB2-C | PV006282 | *Enterovirus B* | CVB2 |
| 2022-338536-E13-B | PV006284 | *Enterovirus B* | E13 |
| 2022-347919-E11-B | PV006287 | *Enterovirus B* | E11 |
| 2022-356900-CVA4-V-1 | PV006288 | *Enterovirus A* | CVA4 |
| 2022-356900-CVA6-V-1 | PV006290 | *Enterovirus A* | CVA6 |
| 2022-356900-CVA6-V-2 | PV006291 | *Enterovirus A* | CVA6 |
| 2022-356900-E11-V | PV006295 | *Enterovirus B* | E11 |
| 2022-356765-CVA19-B | PV006296 | *Enterovirus C* | CVA19 |
| 2022-356765-CVA6-B-1 | PV006298 | *Enterovirus A* | CVA6 |
| 2022-356765-EVD68-B | PV006300 | *Enterovirus D* | EV-D68 |
| 2022-356909-CVB1-V | PV006302 | *Enterovirus B* | CVB1 |
| 2022-367070-CVB5-V | PV006304 | *Enterovirus B* | CVB5 |
| 2022-374107-CVB2-B | PV006306 | *Enterovirus B* | CVB2 |
| 2022-374107-CVB5-B | PV006307 | *Enterovirus B* | CVB5 |
| 2022-374107-E11-B | PV006308 | *Enterovirus B* | E11 |
| 2022-374107-E3-B | PV006310 | *Enterovirus B* | E3 |
| 2022-374107-EVA119-B | PV006311 | *Enterovirus A* | EV-A119 |
| 2022-376079-CVA5-V | PV006312 | *Enterovirus A* | CVA5 |
| 2022-376079-CVB2-V | PV006313 | *Enterovirus B* | CVB2 |
| 2022-376079-E11-V | PV006314 | *Enterovirus B* | E11 |
| 2023-51363-CVA13-C | PV006319 | *Enterovirus C* | CVA13 |
| 2023-51363-CVB5-C | PV006321 | *Enterovirus B* | CVB5 |
| 2023-51363-EVA119-C | PV006322 | *Enterovirus A* | EV-A119 |
| 2023-56206-CVA16-B | PV006323 | *Enterovirus A* | CVA16 |
| 2023-56206-CVB5-B-1 | PV006324 | *Enterovirus B* | CVB5 |
| 2023-56206-E11-B | PV006327 | *Enterovirus B* | E11 |
| 2023-66368-CVA16-V | PV006328 | *Enterovirus A* | CVA16 |
| 2023-66368-E11-V | PV006329 | *Enterovirus B* | E11 |
| 2023-66468-CVA1-V | PV006332 | *Enterovirus C* | CVA1 |
| 2023-66468-E11-V | PV006333 | *Enterovirus B* | E11 |
| 2023-80608-CVA6-B | PV006335 | *Enterovirus A* | CVA6 |
| 2023-80608-CVB2-B | PV006336 | *Enterovirus B* | CVB2 |
| 2023-82310-CVA16-V | PV006337 | *Enterovirus A* | CVA16 |
| 2023-82382-E11-C | PV006340 | *Enterovirus B* | E11 |
| 2023-90342-CVA16-V-1 | PV006341 | *Enterovirus A* | CVA16 |
| 2023-90342-CVA4-V-1 | PV006343 | *Enterovirus A* | CVA4 |
| 2023-90342-E11-V | PV006345 | *Enterovirus B* | E11 |
| 2023-90067-CVA16-C | PV006346 | *Enterovirus A* | CVA16 |
| 2023-90067-CVB5-C | PV006347 | *Enterovirus B* | CVB5 |
| 2023-90371-CVB5-V | PV006348 | *Enterovirus B* | CVB5 |
| 2023-90371-E11-V | PV006349 | *Enterovirus B* | E11 |
| 2023-90371-E18-V | PV006350 | *Enterovirus B* | E18 |
| 2023-99040-E11-V | PV006351 | *Enterovirus B* | E11 |
| 2023-111439-CVA16-B | PV006352 | *Enterovirus A* | CVA16 |
| 2023-111439-CVB5-B | PV006353 | *Enterovirus B* | CVB5 |
| 2023-113496-CVA16-V | PV006356 | *Enterovirus A* | CVA16 |
| 2023-113496-E11-V | PV006357 | *Enterovirus B* | E11 |
| 2023-113537-CVA16-C | PV006358 | *Enterovirus A* | CVA16 |
| 2023-113537-E11-C | PV006359 | *Enterovirus B* | E11 |
| 2023-120564-E11-B | PV006360 | *Enterovirus B* | E11 |
| 2023-120564-EVA76-B | PV006361 | *Enterovirus A* | EV-A76 |
| 2023-122273-CVA10-V | PV006362 | *Enterovirus A* | CVA10 |
| 2023-122273-CVA5-V | PV006364 | *Enterovirus A* | CVA5 |
| 2023-122273-CVB2-V | PV006366 | *Enterovirus B* | CVB2 |
| 2023-122273-CVB5-V | PV006367 | *Enterovirus B* | CVB5 |
| 2023-122273-E11-V | PV006368 | *Enterovirus B* | E11 |
| 2023-122273-E18-V | PV006369 | *Enterovirus B* | E18 |
| 2023-122273-EVC99-V | PV006370 | *Enterovirus C* | EV-C99 |
| 2023-135918-CVA5-V | PV006371 | *Enterovirus A* | CVA5 |
| 2023-135918-CVB5-V | PV006372 | *Enterovirus B* | CVB5 |
| 2023-135918-E11-V | PV006373 | *Enterovirus B* | E11 |
| 2023-135918-E18-V | PV006374 | *Enterovirus B* | E18 |
| 2023-160114-CVB5-B | PV006379 | *Enterovirus B* | CVB5 |
| 2023-161879-CVA16-V | PV006381 | *Enterovirus A* | CVA16 |
| 2023-161879-E11-V | PV006383 | *Enterovirus B* | E11 |
| 2023-161879-EVC99-V | PV006384 | *Enterovirus C* | EV-C99 |
| 2023-168814-CVA5-B | PV006385 | *Enterovirus A* | CVA5 |
| 2023-168814-E11-B | PV006386 | *Enterovirus B* | E11 |
| 2023-170202-E11-V | PV006388 | *Enterovirus B* | E11 |
| 2023-170192-CVA10-C | PV006389 | *Enterovirus A* | CVA10 |
| 2023-170192-E11-C | PV006393 | *Enterovirus B* | E11 |
| 2023-175935-CVB5-V | PV006394 | *Enterovirus B* | CVB5 |
| 2023-175935-E11-V | PV006395 | *Enterovirus B* | E11 |
| 2023-191513-CVA10-B | PV006396 | *Enterovirus A* | CVA10 |
| 2023-191513-CVB5-B | PV006397 | *Enterovirus B* | CVB5 |
| 2023-191536-CVB5-B | PV006398 | *Enterovirus B* | CVB5 |
| 2023-192946-CVA5-C | PV006400 | *Enterovirus A* | CVA5 |
| 2023-192946-CVB5-C | PV006401 | *Enterovirus B* | CVB5 |
| 2023-192992-CVA5-C | PV006402 | *Enterovirus A* | CVA5 |
| 2023-192992-E18-C | PV006403 | *Enterovirus B* | E18 |
| 2023-192992-EVA119-C | PV006404 | *Enterovirus A* | EV-A119 |
| 2023-193112-CVB2-V | PV006406 | *Enterovirus B* | CVB2 |
| 2023-193112-CVB5-V-1 | PV006407 | *Enterovirus B* | CVB5 |
| 2023-193112-E11-V | PV006409 | *Enterovirus B* | E11 |
| 2023-193112-E18-V | PV006410 | *Enterovirus B* | E18 |
| 2023-199926-CVB5-B | PV006413 | *Enterovirus B* | CVB5 |
| 2023-201706-CVA5-C | PV006416 | *Enterovirus A* | CVA5 |
| 2023-326308-CVA13-C | PV006417 | *Enterovirus C* | CVA13 |
| 2023-326308-E11-C | PV006418 | *Enterovirus B* | E11 |
| 2023-353027-CVA1-V | PV006421 | *Enterovirus C* | CVA1 |
| 2023-353027-CVA5-V | PV006422 | *Enterovirus A* | CVA5 |
| 2023-353027-CVA6-V | PV006423 | *Enterovirus A* | CVA6 |
| 2023-353027-E30-V | PV006424 | *Enterovirus B* | E30 |
| 2023-353027-EVC99-V | PV006425 | *Enterovirus C* | EV-C99 |
| 2023-361877-E11-C | PV006426 | *Enterovirus B* | E11 |
| 2023-371672-CVB2-C | PV006427 | *Enterovirus B* | CVB2 |
| 2023-371894-E21-V | PV006428 | *Enterovirus B* | E21 |
| 2023-371894-EVC99-V | PV006429 | *Enterovirus C* | EV-C99 |
| 2023-398531-CVA13-C-1 | PV006430 | *Enterovirus C* | CVA13 |
| 2023-398531-CVA13-C-2 | PV006431 | *Enterovirus C* | CVA13 |
| 2023-398531-CVA6-C | PV006433 | *Enterovirus A* | CVA6 |
| 2023-398531-CVB2-C | PV006434 | *Enterovirus B* | CVB2 |
| 2023-398535-CVA4-V | PV006437 | *Enterovirus A* | CVA4 |
| 2023-398535-E11-V | PV006438 | *Enterovirus B* | E11 |
| 2023-398538-E30-V | PV006441 | *Enterovirus B* | E30 |
| 2023-408427-CVA6-C | PV006444 | *Enterovirus A* | CVA6 |
| 2023-408427-CVB2-C | PV006445 | *Enterovirus B* | CVB2 |
| 2023-413863-E11-V | PV006446 | *Enterovirus B* | E11 |
| 2022-3891-CVA24-V | PV637059 | *Enterovirus C* | CVA24 |
| 2022-3902-CVA22-V | PV637060 | *Enterovirus C* | CVA22 |
| 2022-108668-EVA90-V | PV637061 | *Enterovirus A* | EV-A90 |
| 2022-108668-CVA5-V | PV637062 | *Enterovirus A* | CVA5 |
| 2022-108668-EVA76-V | PV637063 | *Enterovirus A* | EV-A76 |
| 2022-376079-EVA76-V | PV637064 | *Enterovirus A* | EV-A76 |
| 2023-33963-E11-V | PV637065 | *Enterovirus B* | E11 |
| 2023-51377-E11-V | PV637066 | *Enterovirus B* | E11 |
| 2023-82310-E11-V | PV637067 | *Enterovirus B* | E11 |
| 2023-82319-E11-V | PV637068 | *Enterovirus B* | E11 |
| 2023-82319-EVA76-V | PV637069 | *Enterovirus A* | EV-A76 |
| 2023-193112-CVA5-V | PV637070 | *Enterovirus A* | CVA5 |
| 2023-371894-CVA6-V | PV637071 | *Enterovirus A* | CVA6 |
| 2022-188798-CVA6-C | PV637072 | *Enterovirus A* | CVA6 |
| 2023-51363-CVA5-C | PV637073 | *Enterovirus A* | CVA5 |
| 2023-192992-CVA5-C-2 | PV637074 | *Enterovirus A* | CVA5 |
| 2023-201706-E11-C | PV637075 | *Enterovirus B* | E11 |
| 2023-398531-CVA5-C | PV637076 | *Enterovirus A* | CVA5 |
| 2022-347919-EVA76-B | PV637077 | *Enterovirus A* | EV-A76 |
| 2022-356765-CVB5-B | PV637078 | *Enterovirus B* | CVB5 |
| 2022-356765-CVA24-B | PV637079 | *Enterovirus C* | CVA24 |
| 2022-356765-EVA76-B | PV637080 | *Enterovirus A* | EV-A76 |
| 2022-374107-CVA5-B | PV637081 | *Enterovirus A* | CVA5 |
| 2023-111439-EVA76-B | PV637082 | *Enterovirus A* | EV-A76 |
| 2023-199926-CVA5-B | PV637083 | *Enterovirus A* | CVA5 |
